# Signal Processing of MEMS Gyroscope Arrays to Improve Accuracy Using a 1st Order Markov for Rate Signal Modeling

**DOI:** 10.3390/s120201720

**Published:** 2012-02-07

**Authors:** Chengyu Jiang, Liang Xue, Honglong Chang, Guangmin Yuan, Weizheng Yuan

**Affiliations:** Micro and Nano Electromechanical System Laboratory, Northwestern Polytechnical University, 127 Youyi West Road, Xi’an 710072, Shaanxi, China; E-Mails: xueliang@mail.nwpu.edu.cn (L.X.); changhl@nwpu.edu.cn (H.C.); yuangm@nwpu.edu.cn (G.Y.); yuanwz@nwpu.edu.cn (W.Y.)

**Keywords:** MEMS gyroscope array, Kalman filter, first-order Markov process, rate accuracy improvement

## Abstract

This paper presents a signal processing technique to improve angular rate accuracy of the gyroscope by combining the outputs of an array of MEMS gyroscope. A mathematical model for the accuracy improvement was described and a Kalman filter (KF) was designed to obtain optimal rate estimates. Especially, the rate signal was modeled by a first-order Markov process instead of a random walk to improve overall performance. The accuracy of the combined rate signal and affecting factors were analyzed using a steady-state covariance. A system comprising a six-gyroscope array was developed to test the presented KF. Experimental tests proved that the presented model was effective at improving the gyroscope accuracy. The experimental results indicated that six identical gyroscopes with an ARW noise of 6.2 °/√h and a bias drift of 54.14 °/h could be combined into a rate signal with an ARW noise of 1.8 °/√h and a bias drift of 16.3 °/h, while the estimated rate signal by the random walk model has an ARW noise of 2.4 °/√h and a bias drift of 20.6 °/h. It revealed that both models could improve the angular rate accuracy and have a similar performance in static condition. In dynamic condition, the test results showed that the first-order Markov process model could reduce the dynamic errors 20% more than the random walk model.

## 1. Introduction

Micro Electromechanical System (MEMS) gyroscopes have been used for measuring rate or angle of rotation in various inertial measurement fields thanks to their attractive advantages such as small size, low cost, possible batch fabrication and low power consumption [1,2]. However, to date the low accuracy of the MEMS gyroscope has limited its applications to tasks requiring high-precision rate signals. During the development of MEMS gyroscopes, several common methods have been explored to improve the accuracy, mostly focusing on the device itself, such as enhancement in materials, fabrication processes, structural design and interface circuits [3–7]. Other extensive research has been also carried out on the area of accuracy improvement. One of the fundamentally different methods is the virtual gyroscope technology [8], its principle is that several identical low-precision gyroscopes are combined together to form an array and measure the same rate. An optimal rate estimate can be obtained by the optimal filter to fuse these multiple measurements. This approach can further reduce the noise, the bias instability and improve the overall accuracy beyond the performance limitations of individual gyroscopes. Bayard and Ploen constructed a virtual gyroscope using a four-gyro array, the simulation results showed that the gyroscopes with drifts of 8.66 °/h could be combined into a virtual gyroscope with a drift of 0.062 °/h when the gyroscope array are assumed to have a correlation factor of −0.33329 [8]. In our previous work, a MEMS gyroscope array with three individual gyroscopes was studied [9,10], and a two-level optimal filter was designed to compensate the random noises and reduce the bias drift [9]. Al-Majed and Alsuwaidan presented a multi-filters adaptive estimator for the MEMS gyroscope array system to improve the angular rate estimates [11]. Additionally, an extended Kalman filter (EKF) technique for combining multiple sensors was studied in [12].

In recent years, the redundant MEMS inertial sensors have been utilized integrated with GPS to improve navigation performance. Numerous studies and researches have been undertaken on redundant inertial measurement unit (IMU) integration, whereby the measurements of multiple IMUs are fused [13–19], into what can be called a virtual IMU (VIMU). In VIMU theory, the configuration of multiple IMUs is one of the most important considerations. Pejsa presented a theory about how multiple sensors can be placed on a cone to maximize redundancy and mathematically determined the optimal configuration for sensor axes [19]. In [14], an approach for reducing and estimating the noises in the inertial sensors was investigated by using multiple inertial sensors, furthermore the INS/GPS integrator based on the synthetic IMU, extended IMU and geometrically constrained IMU mechanization were presented for integration of GPS with redundant MEMS-IMUs. In [15] the impact of the number of sensors and sensors’ orientation on the system performance were demonstrated, and the optimal configuration of the multiple IMU triads was also studied. Additionally, three mechanization approaches of the system and an observation model for the multiple IMUs integration were described in [16]. Furthermore, in [18] several fusion algorithms were developed using multiple IMUs to enhance performance in the pedestrian navigation. It can be seen that the technology of the virtual gyroscope is essentially identical with the VIMU, since both of them fuse multiple measurements to create a combined signal from a sensor array for improving the overall performance. However, in contrast with the VIMU, the virtual gyroscope usually models the rate signal directly to obtain an optimal rate estimate. Therefore, the objective of this paper, which based on the virtual gyroscope, is to study various models for modeling the rate signal to combine multiple MEMS gyroscopes.

The key of combining multiple gyroscopes for accuracy improvement lies in rate signal modeling and the optimal filter design. Therefore, how to model the rate signal is a prerequisite for constructing a virtual gyroscope system. Through analyzing the current approaches [8–10], it can be found that these approaches usually modeled the rate signal by a random walk and then evaluated performance. However, to model the rate signal as the random walk has some limitations, which will affect the system’s overall performance. The random walk process is a non-stationary process since its variance increases with time; however, in practice most of the rate signal energy is at low frequency. Furthermore, the practical dynamic characteristic of the angular rate cannot always be well represented and reflected by the random walk process in conditions of lacking more information about rate signal such as power-spectral density and bandwidth. The use of random walk models may lead to accuracy degradation, especially signal attenuation in a dynamic situation. To overcome such a problem, the rate signal should be modeled by other suitable approaches.

Markov process modeling is a powerful and commonly used technique that has been introduced for modeling inertial sensors errors for several years, but it has not been used for modeling the virtual gyroscope before. In most applications, the maneuverability characteristics of aircrafts can be regarded as contained in a certain frequency bandwidth and magnitude [20]. In addition, the angular rate signal *ω_k+1_* for the subsequent time point *t_k+1_* usually can be thought as related to *ω_k_* for the former time point *t_k_*. Such dynamic characteristics and property can be suitably represented by a Markov process model. Furthermore, the Markov process is a stationary process that has an exponential Autocorrelation Function (ACF). The ACF of a zero-mean first-order Markov process is defined by a decaying exponential form [21].

Although the dynamic characteristics of the rate signal can be more accurately represented by using an Autoregressive (AR) model of orders higher than one to model the rate signal, it would result in estimating more system parameters and increasing the complexity of filter. On the other hand, the first-order Markov process satisfies the requirement for establishing the system state-space model. Therefore, in this paper the first-order Markov process is used to model the rate signal, and then a complete KF is designed for obtaining the optimal rate estimate. Furthermore, the factors that affect the system performance have been analyzed. Lastly, the hardware of the virtual gyroscope system is implemented and the performance of the virtual gyroscope with two different rate signal models are tested and compared.

## 2. Modeling of Virtual Gyroscope System

**Figure 1.**
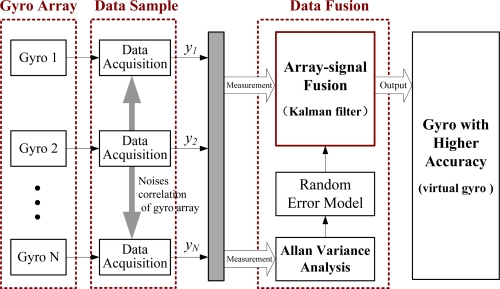
Structure and principle of the virtual gyroscope.

The structure and principle of the virtual gyroscope is shown in Figure 1. Several identical MEMS gyroscopes form a sensor array and the multiple measurements of the sensor array are collected by the data acquisition system. These measurements are used to quantify the random errors through noise modeling by the Allan variance. The optimal filter is designed to combine multiple measurements to produce a minimum variance rate estimate. Therefore, it can provide a combined signal with a higher accuracy than that of the single sensors. The key of the virtual gyroscope lies in the optimal filter, which includes the modeling of rate signal and system state-space. The optimal filter based on the first-order Markov process will be designed. In addition, the KF has the advantage of being a systematic method to ensure minimum variance rate estimation [22]. It is suitable for dealing with dynamic data and has high real-time performance compared with other estimation approach, and widely used to improve performance of the micromechanical inertial sensors [23]. Therefore, the KF is used to implement the gyroscope array.

### 2.1. State Model for Virtual Gyroscope

The true rate signal can be modeled directly to improve accuracy. For most applications of gyroscope, the rate signal is propagated according to an approximate power spectral density function in which the three degree-of-freedom motion is contained. In other words, this motion is expected to be within a certain frequency bandwidth and magnitude [20]. It can be modeled in state space form as a first-order system with a process time constant set according to the system’s bandwidth and the process noise related to the limit of the magnitude of the motion. Therefore, it is suitable to model the true rate signal as first-order Markov process:

(1) $$\dot{\omega }=-1/\tau_{\omega }\cdot \omega +n_{\omega }$$

where *ω* is the true rate signal, *τ_ω_* is the process time constant, *n_ω_* is the zero-mean Gaussian white noise with variance parameter *q_ω_* that can be set according to the dynamic characteristics of the input rate signal. To set the true rate signal as the system state, the state model for the virtual gyroscope system can be established as:

(2) $$\dot{X}(t)=F\cdot X(t)+w(t)=-1/\tau_{\omega }\cdot X(t)+n_{\omega }$$

where system state *X*(*t*) = *ω*, coefficient scalar *F* = −1/*τ_ω_*, system process noise *w*(*t*) = *n_ω_* and *E*[*w*(*t*)*w^T^*(*t*+*τ*)] = *q_ω_δ*(*τ*).

With such direct modeling of the rate signal, the rate signal can be estimated and obtained directly using a KF. Furthermore, the accuracy of the combined rate signal can be analyzed by the KF covariance; this can provide a reference for system improvement and parameters adjustment. In particular, it is suitable for the individual gyroscope which only includes the random noise of the white noise.

### 2.2. Measurement Model for Virtual Gyroscope

The measurement errors of MEMS gyroscope is usually composed of the known errors and random errors. The known errors can be eliminated by the testing procedure, thus only the random errors are discussed for the gyroscope. A common model for the MEMS gyroscope is widely used in many application which mainly includes the white noise denoted as angular random walk (ARW) and bias drift due to rate random walk (RRW) [24,25]. However, for the MEMS gyroscopes with a lower accuracy, the ARW noise is the dominant term rather than RRW; other researchers have made the same observation [26]. In our experience, this is the case for consumer-grade and lower tactical-grade MEMS gyroscopes. Therefore, to estimate and extract the true rate signal from the noise-corrupted measurements and reduce the dominant ARW noise, it is reasonable to model the ARW alone to reduce the dimension of the KF, thus the output rate signal of such gyroscopes can be described as a simplified model:

(3) y(t)=ω(t)+n(t)

where *y*(*t*) is the output rate signal of the gyroscope, *ω*(*t*) is the true rate signal, and *n*(*t*) is the white noise corrupting the gyroscope rate measurement but becoming the angular random walk at the gyroscope angle level. As for a gyroscope array with sensor number of N, the measurement model for the virtual gyroscope system can be expressed as:

(4) Z(t)=H⋅ω+v(t)

and:

(5) $$Z(t)={\left[y_{1},y_{2},\cdots ,y_{N}\right]}^{T},H={\left[1,1,\cdots ,1\right]}_{N\times 1}^{T},v(t)={\left[n_{1},n_{2},\cdots ,n_{N}\right]}^{T}$$

where **v**(*t*) is the measurement noise with *E*[**v**(*t*)] = 0 and *E*[**v**(*t*)**v***^T^*(*t* + *τ*)] = **R***δ*(*τ*), *E*[·] is the mathematical expectation operator, *t* is an arbitrary sampling time and *τ* is the time lag between samples, *δ*(*τ*) is the Dirac delta function.

The matrix **R** is the covariance matrix of the noises **v**(*t*), it will not be necessarily diagonal since the component gyroscopes may be correlated with each other. The correlation between sensor array can be interpreted as the outputs of the component gyroscopes satisfying a statistical relationship. This relationship can be characterized and indicated by a correlation factor *ρ* and covariance matrix **R** [8]. For a gyroscope array with positive correlation factors, the noises of the component gyroscopes will show an identical statistical distribution pattern; negative correlation factors indicate the opposite statistical distribution pattern of the noises. Concretely, correlation factor *ρ =* 0 indicates an uncorrelated gyroscope array; the gyroscope array will have maximum correlation when *ρ =* ±1; when correlation factor −1< *ρ* <1, the gyroscope array will have a correlation in between. Suppose that a constant cross-correlation exists between the identical component sensors, then the covariance matrix **R** for the noises **v**(*t*) can be expressed as:

(6) $$R=\sigma_{n}^{2}\cdot {\left[\begin{array}{cccc} 1 & \rho & \cdots & \rho \\ \rho & 1 & \cdots & \rho \\ \vdots & \vdots & \ddots & \vdots \\ \rho & \rho & \cdots & 1 \end{array}\right]}_{N\times N}$$

where $\sigma_{n}^{2}$ is the ARW noise variance of the component gyroscopes, and *ρ* is the constant cross-correlation factor.

### 2.3. Optimal KF for Angular Rate Estimate

Based on the aforementioned system state-space model of Equations (2) and (4), the continuous KF can be expressed as:

(7) $$\dot{\hat{X}}(t)=F\hat{X}(t)+K(t)[Z(t)-H\hat{X}(t)]$$

(8) $$K(t)=P(t)H^{T}R^{-1}$$

(9) $$\dot{P}(t)=2P(t)F-P^{2}(t)H^{T}R^{-1}H+q_{\omega }$$

Equations (7) to (9) describe the relationship between the system state *X*(*t*), filter gain **K**(*t*) and estimated covariance *P*(*t*). It can be seen that the system state *X*(*t*), *i.e.*, the true rate signal, can be eventually estimated through solving the problem of the differential equation (9), which is called matrix Riccati differential equation (RDE). Therefore, solving the RDE is crucial to KF. From analysis, it indicates that the KF is completely observable due to the rank of observable matrix is equal to the dimension of *X*(*t*), thus the KF will be stable and solution to the RDE will approach a steady-state value, this avoids numerical integration for the RDE and updates *P*(*t*) in each step. For this case, it can get *Ṗ*(*t*) = 0 and Equation (9) goes over into an algebraic equation [27]:

(10) $$2F\cdot P_{\infty }-D\cdot P_{\infty }^{2}+q_{\omega }=0$$

where *P_∞_* is a scalar representing the steady-state covariance of the estimated rate signal, and *D* = **H***^T^***R**^−1^**H**. Since *P_∞_* > 0, solving Equation (10) and the steady-state covariance can be obtained as:

(11) $$P_{\infty }=D^{-1}(-1/\tau_{\omega }+\sqrt{{\left(1/\tau_{\omega }\right)}^{2}+{\mathrm{Dq}}_{\omega }})$$

Inserting Equation (11) into Equation (8) yields:

(12) $$K_{\infty }=D^{-1}(-1/\tau_{\omega }+\sqrt{{\left(1/\tau_{\omega }\right)}^{2}+{\mathrm{Dq}}_{\omega }})H^{T}R^{-1}$$

where **K***_∞_* is a 1 × N row vector representing the steady-state filter gain. Using steady-state gain **K***_∞_*, the rate signal estimate can be propagated in the continuous state-space form:

(13) $$\dot{\hat{X}}(t)=(F-K_{\infty }H)\cdot \hat{X}(t)+K_{\infty }Z(t)$$

**Figure 2.**
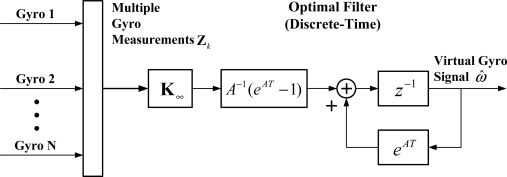
Virtual gyroscope implementation using a discrete KF.

The discrete KF can be derived by directly discretizing the continuous KF of Equation (13) using a zero-order hold approximation. Assume that the measurement value **Z**(*t*) is held constant over each sampling period, we have:

(14) $${\hat{X}}_{k+1}=e^{\mathrm{AT}}{\hat{X}}_{k}+A^{-1}(e^{\mathrm{AT}}-1)K_{\infty }Z_{k}$$

where *A* = −(1/*τ_ω_* + **K***_∞_***H**), and *T* is the sampling period. Consequently, the outputs of the virtual gyroscope can be determined by the discrete KF of Equation (14). The implementation of a virtual gyroscope system makes use of a discrete KF with a structure as shown in Figure 2.

From the above description of state-space model and KF, Equation (2) holds for constant rate input, and Equation (14) can be used to obtain a rate signal with high accuracy for the case of static and constant condition. For dynamic conditions, *i.e.*, for time varying rate inputs, the variance *q_ω_* can be used to set an appropriate bandwidth to satisfy the dynamic requirement.

### 2.4. Performance Analysis of KF

The bandwidth is an important parameter for the KF, it is related to the structure and parameters of the KF such as variance *q_ω_*, number of individual gyroscopes N and noise statistical quantities of the individual gyroscopes. The correlation factor doesn’t affect the bandwidth. From the continuous KF of Equation (13), the transfer function from the outputs of the gyroscope array to virtual gyroscope can be expressed in Laplace transform domain as:

(15) $$\hat{\omega }(s)={\left(s+\sqrt{{\left(1/\tau_{\omega }\right)}^{2}+{\mathrm{Dq}}_{\omega }}\right)}^{-1}K_{\infty }Z(s)$$

Due to a input rate signal is detected by multiple gyroscopes, the outputs of the gyroscope array can be expressed as **Z**(*t*) = **H** · *y_s_*(*t*) without considering the sensors noises, where *y_s_*(*t*) is the output rate signal of the single gyroscope. Using Equations (12) and (15), the transfer function can be formed as:

(16) $$H(s)={\left(s+\sqrt{{\left(1/\tau_{\omega }\right)}^{2}+{\mathrm{Dq}}_{\omega }}\right)}^{-1}(-1/\tau_{\omega }+\sqrt{{\left(1/\tau_{\omega }\right)}^{2}+{\mathrm{Dq}}_{\omega }})$$

The frequency response of the KF can be obtained as:

(17) $$A(\omega )=\frac{\left|H(j\omega )\right|}{\left|H(0)\right|},\;\;A(0)=1$$

The −3 dB standard is used to define the KF bandwidth, using KF frequency response, the bandwidth can be expressed as:

(18) $$\mathrm{BW}=\frac{1}{2\pi }\sqrt{{\left(1/\tau_{\omega }\right)}^{2}+{\mathrm{Dq}}_{\omega }}$$

With the same derivation and analysis, the bandwidth of KF modeled by the random walk can be expressed as:

(19) $${\mathrm{BW}}_{\mathrm{rw}}=\frac{1}{2\pi }\sqrt{{\mathrm{Dq}}_{\omega }}$$

Given the identical system parameters, it is obvious that *BW* is higher than *BW_rw_* and the bandwidth of KF modeled by a first-order Markov is wider than that of the KF modeled by the random walk. Therefore, the presented KF has an improved dynamic characteristic compared to the KF modeled by the random walk.

On the other hand, the performance of the KF and accuracy of the combined rate signal can be evaluated by the steady-state covariance. It can be seen from Equation (11) that the steady-state covariance is affected by three parameters including *D*, *τ_ω_* and *q_ω_*. It indicates that *P_∞_* reduces while decreasing the value of *D*^−1^. Substituting *τ_ω_* → ∞ into Equation (11) results in:

(20) $$P_{\infty }=\sqrt{q_{\omega }/D}$$

It shows that *P_∞_* will be equal to zero when *D*^−1^ = 0, for this case the KF reaches the best performance and the combined rate signal with the best accuracy could be obtained. The *D*^−1^ is related to the number of individual gyroscopes, noise statistical quantities of gyroscopes and correlation factors *ρ*. Using of *D* = **H***^T^***R**^−1^**H**, Equations (6) and (20), the steady-state covariance can be expressed as:

(21) $$P_{\infty }=\sqrt{q_{\omega }\sigma_{n}^{2}\frac{1+(N-1)\rho }{N}}$$

**Figure 3.**
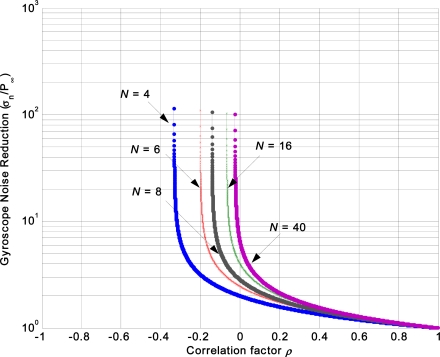
Relationship between the gyroscope noise reduction and correlation factor *ρ* and sensors number N.

Therefore a minimum *P*_∞_ can be achieved through adjusting the factors in Equation (21). In particular, *P*_∞_ will approach zero as *ρ* → −1/(*N*−1). Setting parameter *q_ω_* = 1 in Equation (21), we obtain $\sigma_{n}/P_{\infty }=\sqrt{N/[1+(N-1)\rho ]}$. The relation between the gyroscopes noise reduction, correlation factor *ρ* and sensor number N is shown in Figure 3. It indicates that the magnitude of noise reduction increases with decreasing of factor *ρ*, and the reduction magnitude with a negative correlation is higher than that of a positive one. Furthermore, the correlation factor (either positive or negative) cannot degrade the individual gyroscope accuracy.

In addition, Equation (21) shows that *P*_∞_ will decrease when *q_ω_* is decreased. As for Equation (1) for modeling rate signal, the *q_ω_* can be chosen as it depends on the true rate signal characteristics. In a practical situation, the characteristics of the true rate signal cannot be accurately obtained because of dynamic behaviors of the input rate signal. However, from Equation (18), it can be seen that the KF bandwidth is related to the variance *q_ω_*. Consequently, the KF bandwidth can be used to choose an appropriate value of *q_ω_* to implement the virtual gyroscope system, that is, the requirement of various dynamic applications can be satisfied by adjusting *q_ω_* to obtain different bandwidths.

On the other hand, different values of *q_ω_* will lead to different system performances. Due to the actual statistical model of the rate signal is difficult to accurately obtain, and even *q_ω_* varies with changes of the environment, thus if the choice of *q_ω_* could accurately or closely reflect the dynamic characteristics of the input rate signal, the KF will reach the best performance and the virtual gyroscope signal with the best accuracy can be obtained; while *q_ω_* is smaller than such ‘value’, the performance of the KF will be degraded, it would result in a signal attenuation; while *q_ω_* is higher than such ‘value’, the performance of the KF will be also degraded and eventually reach the level of a simple averaging process with increasing of *q_ω_*.

## 3. Experiments and Discussion

### 3.1. Correlations between the MEMS Gyroscope Array

The performance of the KF was analyzed in Section 2.4. It showed that the accuracy could be considerably improved when the gyroscope array has a negative correlation. However, in a practical implementation, the gyroscope array with an expected correlation factor has so far been hard to artificially design and fabricate. Additionally, usually the performance can be further improved through increasing the number N of the individual sensors in the array (Figure 3); however, one needs to consider the system complexity and uniformity between the gyroscopes. Therefore, in this work, six separate identical ADXRS300 MEMS gyroscopes are utilized to form a gyroscope array to serve as the virtual gyroscope system.

The correlation matrix can be used to indicate noise correlation between the multiple gyroscopes. Since the individual gyroscopes are described by a simple model of Equation (3), the component elements of the correlation matrix, *i.e.*, correlation factors between every pair of sensors, can be obtained by:

(22) $$\mathrm{CorrM}(i,j)=\frac{C(g_{i},g_{j})}{\sqrt{C(g_{i},g_{i})C(g_{j},g_{j})}},i,j=1,2,\cdots ,6$$

where **CorrM** is a 6 × 6 correlation matrix of gyroscope array, *C = cov*(*g_i_*, *g_j_*) is the covariance between the outputs noise of the *i*th and *j*th gyroscope. The correlation matrix of the six-gyroscope array was analyzed with a zero rate output recorded for 1 hour at a sampling rate of 200 Hz. Through multiple tests, the correlation matrix illustrated in Table 1 was obtained, where it is obvious that the correlations between the separate gyroscopes are relatively weak and values of correlation factor are close to zero, therefore, such a gyroscope array can be regarded as uncorrelated. It is reasonable to suppose that the main reason lies in the independent working condition for the mechanical sensing elements and controlling interface circuits of the individual gyroscopes [9].

### 3.2. Hardware Implementation

**Figure 4.**
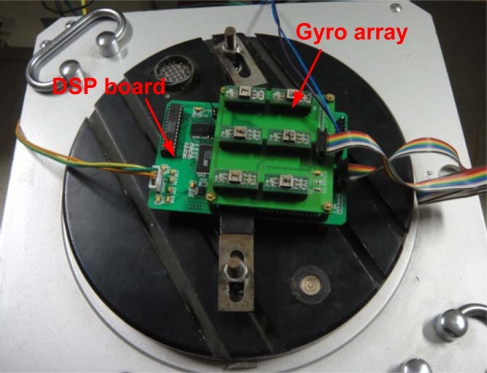
A prototype of the virtual gyroscope system.

The hardware of the virtual gyroscope is mainly composed of sensor array, central processing unit, A/D data acquisition unit, serial communication unit and FLASH memory. Figure 4 shows the prototype of the virtual gyroscope system.

Six ADXRS300 MEMS gyroscopes are utilized to form a gyroscope array. A TMS320VC5416 DSP chip is chosen as core processor for the virtual gyroscope. The A/D data acquisition unit uses a 16-bit ADS7807 to collect voltage signals from gyroscope array. The system operates in serial mode and connects with DSP directly through the 16-bit data bus. FLASH memory unit uses 4Mbit Flash chip AM29LV400 to provide storage space for external system program. The virtual gyroscope signal is exported by RS-232. The experimental results will be presented to quantify the performance of the virtual gyroscope. The geometry configuration of the sensors array should be considered to improve the estimation process. It had been shown in [16,18] that the separation between two sensors will introduce an angular acceleration that relates the individual states through differential equations in fusing redundant IMUs. As the focus of this paper is not on the geometry layout of the gyroscope array, the influence of sensors geometry on the system performance will be investigated in the successive work.

### 3.3. Static Performance Test

**Figure 5.**
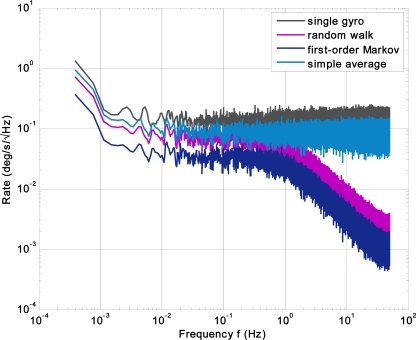
FFT plot of the virtual gyroscope compared to the single gyroscope and averaging outputs of the gyroscope array.

**Figure 6.**
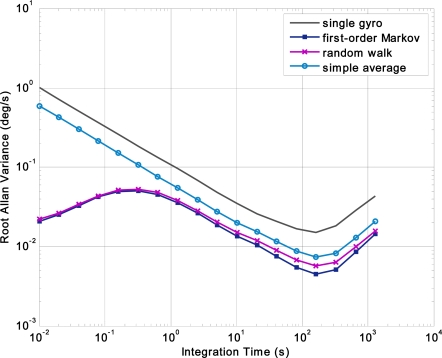
Allan variance results of the virtual gyroscope compared to the single gyroscope and averaging outputs of the gyroscope array.

The ARW, bias drift and noise density of the virtual gyroscope with two KF models are tested and compared. The performances of the virtual gyroscope are evaluated using FFT analysis and root Allan variance of a zero rate output recorded for 1 hour. Due to the 40 Hz bandwidth of an individual gyroscope, and the bandwidth of the virtual gyroscope will not be higher than 40 Hz, thus the sampling rate was set to 200 Hz to satisfy the Nyquist theorem. The system parameters are chosen as *τ_ω_* = 500 s and *q_ω_* = 0.0772 deg^2^/s^3^. The comparisons of FFT plot and Allan variance measurement between the virtual gyroscope and single gyroscope are shown in Figures 5 and 6, respectively. Furthermore, the accuracy of the virtual gyroscope is compared with the averaging outputs of six individual gyroscopes. The detailed results are illustrated in Table 2.

From the FFT plot, the noise level indicates noise floor of ∼0.11°/s/√Hz for the single gyroscope and 0.03°/s/√Hz for the virtual gyroscope modeled by the first-order Markov process, whereas the corresponding value estimated by the random walk model is 0.04°/s/√Hz. It also suggests a low-pass characteristic of the KF. In addition, it indicates a noise floor of ∼0.05°/s/√Hz for the rate signal obtained by averaging outputs of the six individual gyroscopes.

From the Allan variance plot, both the ARW noise and bias drift are reduced by fusing multiple measurements from gyroscope array. Table 2 reveals that the ARW noise of the single gyroscope is reduced from 6.17 °/√h to 1.83 °/√h and 2.35 °/√h by two different models, indicating an ARW reduction factor of about 3.4 by the first-order Markov model. Meanwhile, the bias drift is reduced from 54.11 °/h to 16.32 °/h, which is lower than that of the 20.65 °/h estimated by the random walk model. It is clear that the performances of two models are comparable due to the static characteristics of the angular rate signal. Furthermore, the Allan variance results reveal that values of the ARW noise and bias drift for rate signal estimated by the simple averaging are 2.73 °/√h and 22.72 °/h, respectively, which are greater than that of the presented KF, thus the performance of the presented KF is higher than that of the simple averaging process. Additionally, it can be seen that the ARW reduction is greater than that of the RRW and bias drift, the main reason being that the ARW is the dominant noise in the single gyroscope and the virtual gyroscope adopts a simple error model to describe the relationship of the input angular rates and gyroscope outputs.

### 3.4. Dynamic Performance Test

The dynamic tests are carried out on a horizontal turntable (Figure 4). The standard deviation (1σ) of the estimated errors is used to evaluate the accuracy of the rate signal before and after KF. The sampling rate and parameter *q_ω_* are set to 500 Hz and 1.9290 deg^2^/s^3^.

**Figure 7.**
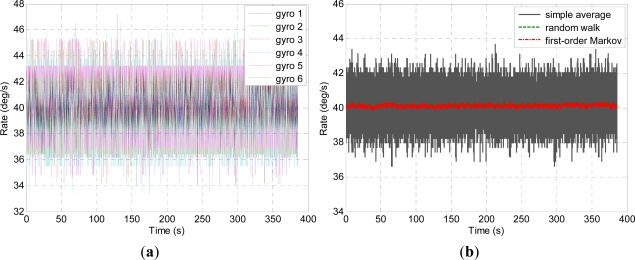
Constant rate test of the virtual gyroscope. (**a**) Outputs of the individual gyroscopes; (**b**) Outputs of the virtual gyroscope.

**Figure 8.**
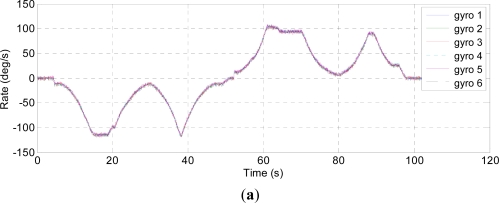

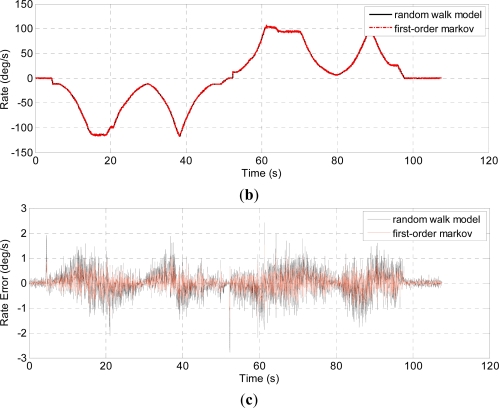
Random rate test of the virtual gyroscope. (**a**) Outputs of the individual gyroscopes; (**b**) Outputs of the virtual gyroscope; (**c**) Estimated rate errors.

**Figure 9.**
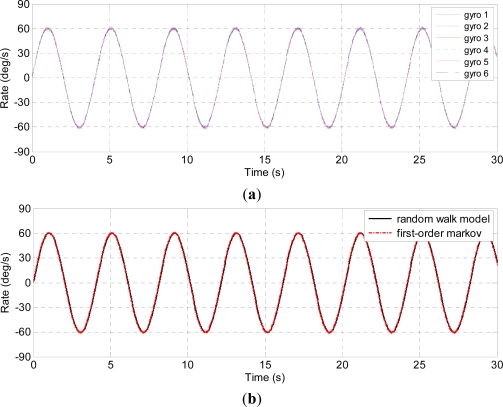

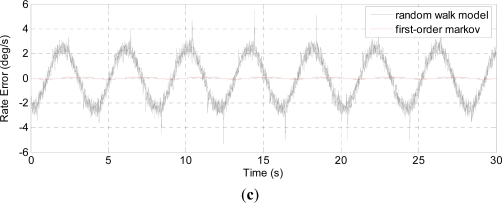
Sinusoidal rate test of the virtual gyroscope. (**a**) Outputs of the individual gyroscopes; (**b**) Outputs of the virtual gyroscope; (**c**) Estimated rate errors.

Three kinds of dynamic condition are chosen to test the presented model. Firstly, the turntable is controlled to rotate in the horizontal plane with a 40 °/s constant rate, the outputs of the individual gyroscopes and virtual gyroscope are shown in Figure 7. Secondly, the turntable is controlled to rotate with a random input rate, the outputs of the individual gyroscopes and virtual gyroscope are shown in Figure 8. Lastly, the turntable swings with the 40° angle amplitude and 0.25 Hz frequency, thus the input rate signal is *ω =* 62.8 × *sin*(1.57*t*) °/s, the results of the virtual gyroscope are shown in Figure 9, here only the data of the first 30s are demonstrated to display the results more clearly. The detailed results are illustrated in Table 3.

Table 3 shows that the gyroscopes’ 1σ errors are reduced from 1.45 °/s to 0.06 °/s and 0.05 °/s by two different models in the constant rate test. It can be seen that the performance improvement of two different models are nearly equivalent. In addition, from Figure 7, the bound on the angular position error caused by the ARW and RRW noisy terms can be obtained by integrating the outputs of gyroscopes over time [28].

In the random rate test, the virtual gyroscope signals could well reflect the dynamic characteristic of the input angular rate (Figure 8), the 1σ errors are reduced from 1.61 °/s to 0.47 °/s and 0.29 °/s, respectively, by both different models. It reveals that the performance of the first-order Markov process model is higher than that of the random walk due to the input rate signal has a more dynamic characteristic than constant condition.

In the swing test, the amplitude of combined rate signal estimated by the first-order Markov model reaches to 62.29 °/s, which is basically in accordance with the experimental setting, meanwhile, the 1σ errors are reduced to 0.16 °/s that is much smaller than 1.8 °/s estimated by the random walk model. Furthermore, Figure 9(c) indicates amplitude attenuation by the random walk model, and the virtual gyroscope signal cannot accurately reproduce the dynamic characteristic of the input rate signal.

It would be useful to verify the presented KF to expand the experiments to different axes and rotations sensed by several axes in addition to the experiments. However, such experiment is difficult to conduct because of the limitation in testing condition and all the individual gyroscopes are oriented along the same axis. Therefore, the verification in the paper was only implemented through such experiments.

## 4. Conclusions

In this paper, the first-order Markov process was used to model the rate signal for fusing multiple MEMS gyroscopes to improve the overall accuracy. It indicated that the six-gyroscope array with an ARW noise of 6.17 °/√h and a bias drift of 54.11 °/h were combined into a rate signal having an ARW noise of 1.83 °/√h and a bias drift of 16.32 °/h. The presented KF also reduced the dynamic errors by over 20% compared to the KF modeled by the random walk. It proved that the first-order Markov process is efficient for modeling rate signal to improve the system overall performance.

In the future fabrication of a number of integrated MEMS gyroscope arrays on a single chip would enhance the uniformity between the gyroscopes and the correlation between the gyroscope array still need to be further researched and explored.

## Figures and Tables

**Table 1.** Correlation matrix of noises for six-gyro array.

	**Gyro1**	**Gyro2**	**Gyro3**	**Gyro4**	**Gyro5**	**Gyro6**
**Gyro1**	1.000	0.021	0.007	0.005	0.005	0.004
**Gyro2**	0.021	1.000	0.038	0.016	0.007	0.009
**Gyro3**	0.007	0.038	1.000	0.036	0.015	0.009
**Gyro4**	0.005	0.016	0.036	1.000	0.038	0.017
**Gyro5**	0.005	0.007	0.015	0.038	1.000	0.032
**Gyro6**	0.004	0.009	0.009	0.017	0.032	1.000

**Table 2.** Static test results of the virtual gyroscope.

**Terms**	**Single gyroscope**	**Simple average**	**Virtual gyroscope performance**	
			**Random walk**	**1st order Markov**
**Noise Density(°/s/√Hz)**	0.11	0.05	0.04	0.03
**ARW (°/√h)**	6.17	2.73	2.35	1.83
**RRW (°/h/√h)**	294.28	161.21	125.83	120.61
**Bias Drift (°/h)**	54.11	22.72	20.64	16.32

**Table 3.** Dynamic test results of the virtual gyroscope (unit: °/s).

**Model**	**Constant rate**		**Random rate**	**Sinusoidal rate**	

	**Mean**	**1σ**	**1σ**	**Amplitude**	**1σ**
**Single gyroscope**	40.15	1.45	1.61	62.64	0.79
**Random walk**	40.14	0.06	0.47	60.47	1.80
**1st order Markov**	40.09	0.05	0.29	62.29	0.16
